# Development of monoclonal antibodies against SARS-CoV-2 nucleocapsid protein for COVID-19 antigen detection

**DOI:** 10.1186/s41182-025-00756-y

**Published:** 2025-05-13

**Authors:** Maurine Mumo Mutua, Bernard N. Kanoi, Steven Ger Nyanjom, Sebastian Musundi, Mark Makau, Shingo Inoue, Samoel Ashimosi Khamadi, Jesse Gitaka, Ernest Apondi Wandera

**Affiliations:** 1 Department of Biochemistry, Jomo Kenyatta University of Agriculture and Technology, Nairobi, Kenya; 2 Centre for Research in Infectious Diseases, Directorate of Research and Innovation, Mount Kenya University, P.O. Box 342-01000, Thika, Kenya; 3 Kenya Medical Research Institute, Graduate School of Health, Nairobi, Kenya; 4 Institute of Tropical Medicine, Nagasaki University-KEMRI, Nairobi, Kenya; 5 Centre for Virus Research, Kenya Medical Research Institute, Nairobi, Kenya; 6 Innovation and Technology Transfer Division, Kenya Medical Research Institute, Nairobi, Kenya

**Keywords:** Monoclonal antibodies, SARS-CoV-2, QueryNucleocapsid protein, COVID-19, Diagnostics

## Abstract

**Background:**

The coronavirus disease 2019 (COVID-19) pandemic underscored the global need for reliable diagnostic tools with quick turnaround time for effective patient management and mitigation of virus spread. This study aimed to express severe acute respiratory syndrome coronavirus 2 (SARS-CoV-2) nucleocapsid protein and produce monoclonal antibodies (mAbs) against the expressed protein.

**Methods:**

Following successful expression and purification of His-tagged SARS-CoV-2 N protein using a wheat germ cell-free protein expression system (WGCFS), BALB/c mice were immunized, and generated hybridomas screened for mAb production. Indirect and sandwich ELISA were used to screen the reactivity of the monoclonal antibody against both our recombinant antigen and commercial antigen. The mAbs were also assessed for their performance using RT-PCR confirmed positive samples with varying cycle threshold (CT) values and their specificity screened using virus isolates of other respiratory viruses.

**Results:**

Our mAb demonstrated high reactivity against our recombinant antigen, commercial antigen, SARS-CoV-2 Beta and Omicron variants. There was no significant difference in the binding affinity of our mAb and commercial mAb against the study recombinant (p = 0.12) and commercial (p = 0.072) antigens. Our mAb detected SARS-CoV-2 from clinical samples with varying CT values and exhibited no cross-reactivity against other respiratory viruses.

**Conclusions:**

We successfully expressed SARS-CoV-2 N protein leveraging WGCFS in a resource-limited setting. Our mAb had a high binding affinity to the recombinant antigen, making it a suitable candidate for antigen detection kit development. Beyond diagnostics, the mAb holds potential for therapeutic applications as well as use in clinical and environmental surveillance platforms.

## Introduction

The severe acute respiratory syndrome coronavirus 2 (SARSCoV2) genome encodes four structural proteins, namely, Spike (S), Membrane (M), Envelope (E) and Nucleocapsid protein (N) [1]. The N and S proteins are the key targets for diagnosis and serological surveillance [2]. The N protein serves as an antigen regulating RNA replication and mRNA transcription, while the S protein plays a key role in viral entry into the host cell by binding to the human angiotensin-converting enzyme 2 (ACE-2) to initiate infection [3]. While the S protein has more than 30 mutations in the currently circulating Omicron variant of concern [4], the N protein remains relatively conserved and stable and, therefore, a key target for diagnostic testing [5]. Furthermore, anti-N antibodies can be used to differentiate between SARS-CoV-2 in natural infection and vaccine-induced antibody response via the S protein [5].

Reverse transcription polymerase chain reaction (RT-PCR) is the gold standard for COVID-19 diagnosis and the most used technique for detecting active infection [6]. RT-PCR accuracy depends on several factors including timing of sample collection, sample processing, technical expertise, long turn-around time and sample storage [7]. Enzyme-linked immuno-assays (ELISA) such as IgM and IgG are based on antibody production after infection and, therefore, not suitable for acute detection as antibody generation takes time [8]. Antigen detection kits are reliable serological tests that detect active infections, even in individuals who are asymptomatic, and are suitable for mass testing as they are affordable, have a quick turnaround time, and can be used in resource-limited settings [9]. The effectiveness of an antigen-detection kit primarily depends on the use of high-quality monoclonal antibodies (mAbs) that accurately target specific viral antigens [10].

Muromonab-CD3 was the first mAb to be approved for clinical use by the US Food and Drug Administration (FDA) in 1986 [11]. Since then, mAbs have been licensed yearly with an estimated US$75 billion sales as of 2021 [11]. mAbs are highly specific and reliable reagents that have been applied in many molecular and immunological investigations [10]. They have become essential reagents for detection [12–14] as well as treatment [15, 16] of various diseases owing to their specificity and versatility, as they are produced from a stable cloned hybridoma [14]. mAbs are produced in human form for immunotherapy to avoid adverse reactions, while for immunodiagnostics, mAbs are produced in mice using hybridoma technology [17].

Neutralizing mAbs (NMAbs) have been produced against different coronaviruses. During the SARS-CoV-1 outbreak in 2002/2003, CR3014 and CR3022 NMAbs were identified which, when combined, effectively neutralized multiple SARS-CoV-1 variants. In addition, B1, the first S2-targeting mAb, was discovered from convalescent patient antibody libraries, providing a strategy for targeting conserved viral regions [18]. NMAbs m336, m337, m338, 3B11, MERS-4, and MERS-27 were identified from phage- and yeast-displayed libraries from healthy donors and effectively blocked Middle East Respiratory Syndrome Coronavirus (MERS-CoV) RNA binding domain (RBD) binding to Dipeptidyl Peptidase 4 (DPP4). In addition, MCA1, derived from MERS-CoV-infected patients, also targeted the RBD, inhibiting viral invasion [19, 20]. NMAb S309 recovered from the memory B cells of SARS-CoV-1 an infected patient during the 2003 outbreak has shown cross-neutralization reactivity against both SARS-CoV-1 and SARS-CoV-2 [18]. In addition, COVID-19 convalescent sera exhibited cross-reactivity to the MERS-CoV S2 subunit, likely due to structural similarities, suggesting that convalescent sera could aid in developing mAbs targeting multiple coronaviruses [21]. Bamlanivimab and etesevimab are among the anti-spike SARS-CoV-2 mAbs approved for early therapy, while tixagevimab and cilgavimab were approved for pre-exposure prophylaxis in 2021 by FDA during the COVID-19 pandemic [11].

As COVID-19 becomes endemic and continues to cause periodic outbreaks, alongside other circulating respiratory viruses with overlapping symptoms, the use of accurate diagnostic tools is essential for effective patient management and reliable disease surveillance. In response to the COVID-19 pandemic, monoclonal antibodies against different SARS-CoV-2 proteins were used to develop antigen kits for detection of SARS-CoV-2 [10, 22]. The quality of antigen used in immunization plays a critical role in determining the precision of mAbs produced [10]. Cell-free protein expression provides a rapid and efficient approach to producing high-quality proteins while maintaining their structure and functionality [23].

Wheat germ cell free system (WGCFS) is an excellent alternative to mammalian cell expression for proteins requiring rapid and scalable production [24]. WGCFS has been demonstrated to produce near native and functionally active proteins while negating posttranslational protein modifications that reduce antigenicity. In addition, the system can express membrane proteins in liposomes, which are extremely difficult to express in classical systems and have previously been used to express more than 2000 proteins from the complex *P. falciparum* genome [25, 26]. In addition, a study that expressed RNA binding domain protein for SARS-CoV-2 using WGCFS confirmed the protein retained proper conformation and receptor-binding capabilities, making it suitable for therapeutic research and serological assays [23].

In this study, we leveraged the WGCFS to synthesize viral antigens to produce mAbs against SARS-CoV-2 N protein in a resource-limited setting. Using the generated mAbs, we detected the N-antigen generated from commercial antigen in a concentration-dependent manner. In addition, our mAbs were able to detect both the beta and omicron variant.

## Materials and methods

### Target gene amplification

Clinical samples confirmed positive for SARS-CoV-2 by real time PCR (RT-qPCR) were used for N gene amplification. Briefly, cDNA synthesis was performed using the ReverTra Ace® qPCR RT Kit (Toyobo) following the manufacturer's instructions. The resulting cDNA (Wuhan-Hu-1, GenBank MN908947.3) was subjected to PCR amplification using specific primers targeting the N gene: (forward primer: 5'-GAGAGAGACTCGAGCGAGGACAAGGCGTTCCAATTAAC-3’ and reverse primer: 5'-GAGAGAGAGCGGCCGCTTATTTTTCAAACTGCGGATGGCTCCAGGCCTGAGTTGAGTCAGCACTGC-3'). KOD-Plus-Ver.2 high-fidelity DNA polymerase kit (Toyobo) was used for amplification according to the manufacturer's protocol. Amplicons were visualized by 1.2% agarose gel electrophoresis and purified using the Wizard® SV Gel and PCR Clean-Up System (Promega) following the manufacturer's instructions.

### Recombinant protein expression and purification

The purified PCR amplicons were digested with restriction enzymes (NotI and XhoI, New England Biolabs®), and ligated into a pEU–E01–His–TEV–MCS, a WGCFS-specific protein expression plasmid. Positive clones were confirmed by colony PCR and Sanger sequences. The sequenced fragments were blasted against non-redundant databases in NCBI blast to confirm the cloned N gene and aligned against the SARS-CoV-2 N gene reference (Wuhan-Hu-1, GenBank MN908947.3) genome using MAFFT to identify any mutations that might introduce stop codons within the coding region of the antigen. This ensured that the expressed protein retained its complete amino acid sequence. The sequences were also aligned against Africa and Kenya specific sequences.

Protein expression was performed using WGCFS as previously described [27]. Briefly, plasmid-derived mRNA was synthesized using SP6 RNA polymerase and translated via the bilayer method [23, 28]. For transcription, a total mixture of 200 µl containing 40 µl of 5X transcription buffer, 20ul of 25 mM nucleotide triphosphates mixture, 2.5 µl of SP6 polymerase (80U/µl), 2.5 µl of RNase inhibitor (80U/µl), 20 µl of plasmid DNA and 115 µl of nuclease-free water was incubated at 37 °C for 18 h. The reaction was centrifuged at 15,000 rpm for 15 min and the supernatant containing the mRNA subjected to translation. Using bilayer translation, a lower layer consisted of 250 µl of 240 OD/ml wheat germ extract (Cell Free Sciences, Yokohama, Japan), mRNA and 20 mg/mL of creatine kinase, while the upper layer contained 5.5 ml of Sub-AMIX buffer made up of 30 mM HEPES, pH 7.8, 100 mM potassium acetate, 2.7 mM magnesium acetate, 1.2 mM ATP, 0.25 mM GTP, 16 mM creatine phosphate, 0.4 mM spermidine, 0.3 mM of each amino acid, 24 units of RNase inhibitor, and 60 μg of liposomes. The upper layer was poured into a plate well, while the lower layer was drawn into a micropipette tip, ensuring no air remained at the tip's end. The tip was then carefully inserted to the bottom of the well containing the upper layer. The mixture was gently dispensed under the buffer without mixing, avoiding the formation of bubbles, so that the upper and lower layer formed a bilayer. The samples were not mixed, and the plate was handled without shaking and was incubated at 16 °C for 72 h. The resulting protein was purified using affinity chromatography. Briefly, a column with Ni-Sepharose Fast Flow beads (Cytiva, Waukesha, WI, USA) was used as stationary phase in the presence of imidazole. Our target protein was bound to the Ni-Sepharose ligand, while the other wheat germ proteins were washed using phosphate buffer and the pure protein eluted using elution buffer. The protein purity and molecular weight were assessed through SDS–PAGE (Coomassie staining, CBB Plus) and western blot.

### Production and characterization of monoclonal antibodies against SARS-CoV-2 N protein

**Fig. 1:**
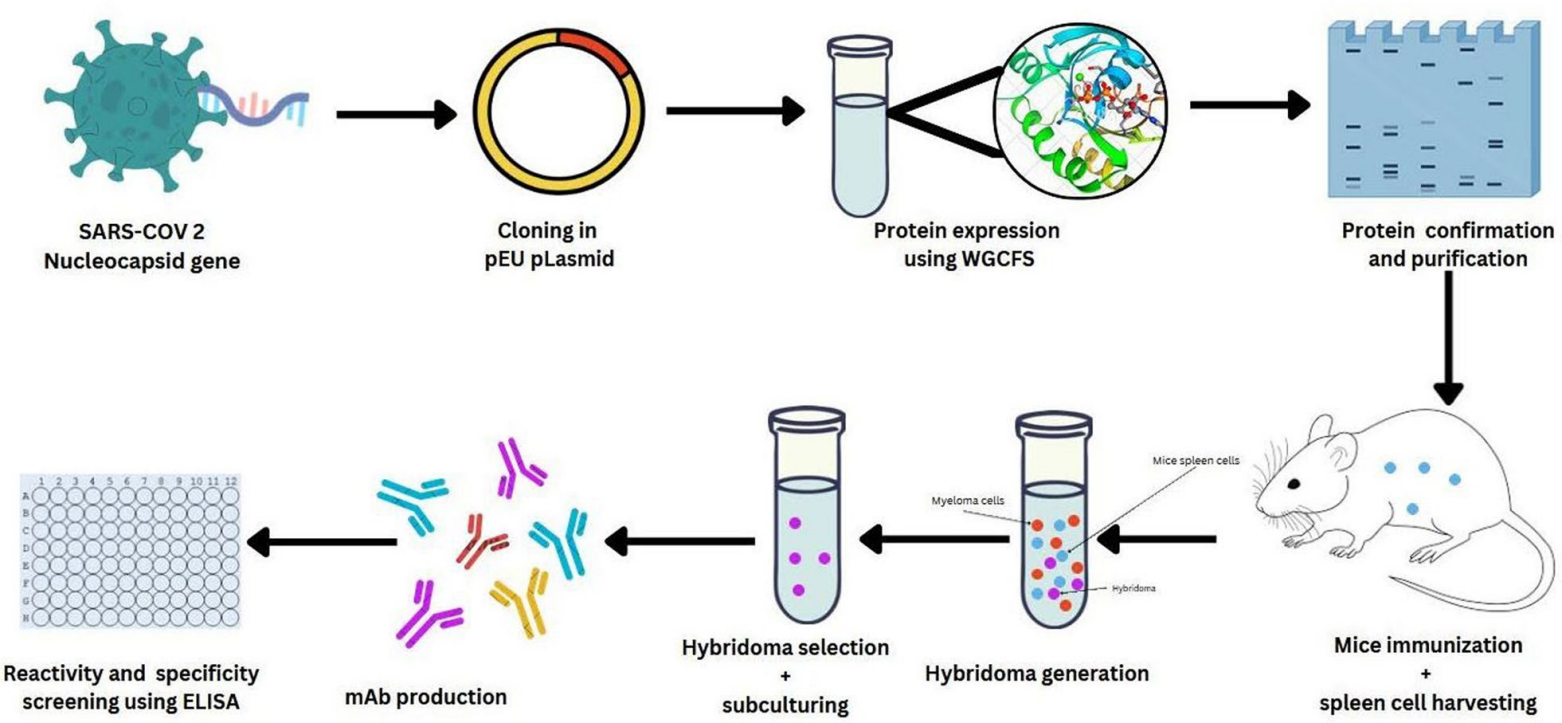
Summary of monoclonal antibody production against SARS-CoV-2 NP using wheat-germ cell free system

BALB/c mice were immunized with purified recombinant SARS-CoV-2 N protein (50 µL subcutaneously and 100 µL intraperitoneally) with Ribi (sigma) adjuvant after 2 week interval. To generate hybridomas, spleen cells were fused with SP 2/0 myeloma cell line (ATCC#CRL-1581) with polyethylene glycol 1500 (PEG 1500) and cultured in HAT-1640 medium containing Hypoxanthine (H), aminopterin (A), and thymine (T). To select stable hybridomas, we employed indirect ELISA against our recombinant N protein. Briefly, recombinant SARS-CoV-2 N protein (1 µg/ml) was used as the coating antigen for 96-well plates and incubated overnight at 4 °C. After blocking using 2% bovine serum albumin (BSA), 20 hybridomas were diluted from 1:10^1^ to 1:10^8^ starting with 1 mg/ml and incubated for 1 h at 37 °C. After three washes with PBS-T, the wells were incubated with 100 µl of HRP–anti-mouse IgG antibody (1:5,000) for 1 h. After three washes with PBS-T, 100 µl of OPD substrate (O-phenyl diamine) was added to the wells and incubated in the dark for 30 min. 100 µl of 1 N sulfuric acid was used to stop the reaction and absorbance read at 492 nm. Five hybridomas with the highest absorbance indicating strong antibody binding were selected for sub-cloning to select stable hybridoma. Indirect ELISA was used to select the most reactive hybridoma, which was cultured in-vitro for mAbs production. The produced mAbs were purified using affinity chromatography and characterized using ELISA. The summary of mAbs production is illustrated (Fig. 1).

### Indirect ELISA for reactivity screening

A 96-well plate was coated with our recombinant SARS-CoV-2 N antigen (CoVAT) and commercial recombinant SARS-CoV-2 N antigen (Nagasaki University, DIA-5028) concurrently except for blank. Following blocking and washing to prevent nonspecific binding, our mAb and commercial mAb (2G4–H1–B2–C3, Cat No. 130–10836) were added to the wells to separately with varying concentration from 2 µg/ml to 0.03125 µg/ml and incubated at 37 °C for 1 h. 100 µl of HRP-conjugated anti-mouse IgG was added (1:5,000) into the wells and incubated at 37 °C for 1 h. The reaction was visualized using an OPD substrate and absorbance read at 492 nm. This method assessed the binding specificity and strength of our mAb against the recombinant and commercial antigens. It also compared the binding affinity of our mAb and commercial mAb.

### Sandwich ELISA for reactivity screening against SARS-CoV-2 variants

The mAb reactivity with SARS-CoV-2-infected fluid culture (ICF) derived from the Beta and Omicron variants was evaluated. Plate-1 was coated with our mAb in two folds serial dilutions and incubated overnight at 4 °C. After blocking and washing as previously described. Virus isolates (ICF) of 3.45 × 10^5^ plague forming unit/ml (PFU/ml) of Beta and Omicron variants were added to the plate in duplicates and incubated at 37 °C 1 h. Concurrently, Plate-2 was prepared by forming a complex of commercial mAb with HRP-conjugated anti-mouse IgG, which was then transferred to Plate-1, incubated at 37 °C for 1 h. Culture media with no viruses were used as a negative control. After incubation and washing with PBST, the reaction was detected using an OPD substrate and absorbance read at 492 nm. This approach minimized cross-reactivity between the coated mAbs and the secondary antibody without SARS-CoV-2 ICF, ensuring specificity in detecting the target antigen.

### Screening of mAb for clinical performance using ELISA

A 96-well plate was coated with our mAb (1 µg/ml) except for the blank well and incubated overnight at 4 °C. Following blocking and washing as previously described, RT-PCR SARS-CoV-2 confirmed nasopharyngeal swabs with varying CT values ranging from 15.45 to 31.18 were added to respective wells and incubated at 37 °C for 1 h. A known SARS-CoV-2 negative sample was used as a negative control. Following washing with PBST, a mouse anti-SARS-CoV-2 HRP conjugated IgG secondary antibody was added to all wells except the blank. After incubation and washing, OPD substrate and stop solution were added as previously described and absorbance read at 492 nm.

### Screening the mAbs specificity using ICF of respiratory viruses

Sandwich ELISA was used to screen for mAbs specificity. Briefly, our mAb (1 µg/ml) was coated in a plate and incubated overnight at 4 °C. After blocking and washing, virus isolates of Human parainfluenza virus (HPIV-1,2,3), Adenovirus (Adv), A-pH1 N1—influenza A virus type H1 N1, A-H3—influenza A virus type H3 N2, Influenza B virus (BS), B/Yamagata lineage, Influenza B virus (BB), B/Victoria lineage, Respiratory syncytial virus (RSV), Herpes simplex virus (HSV type 1/2), SARS-CoV-2 Beta and Omicron mutants were added in triplicates to the coated plate in designated wells. SARS-CoV-2 N antigen was used as a positive control, while culture medium with no virus was used as negative control. After 1 h of incubation and washing at 37 °C, a complex of commercial mAb with HRP-conjugated anti-mouse IgG was added and incubated for 1 h. After washing, the reaction was detected using OPD substrate and absorbance determined at 492 nm. The mean absorbance of three independent assays was calculated.

### Statistical analysis

All plots and statistical analyses were conducted using R software v4.4.1. Inferential statistical methods (student *T* test or Kruskal–Wallis test) compared whether there was a significant difference in the reactivity of the study mAb versus a commercial mAb with a *p* value < 0.05 showing statistical significance.

## Results

### Wheat germ cell free system allows for generation of high-quality recombinant N-protein

**Fig. 2:**
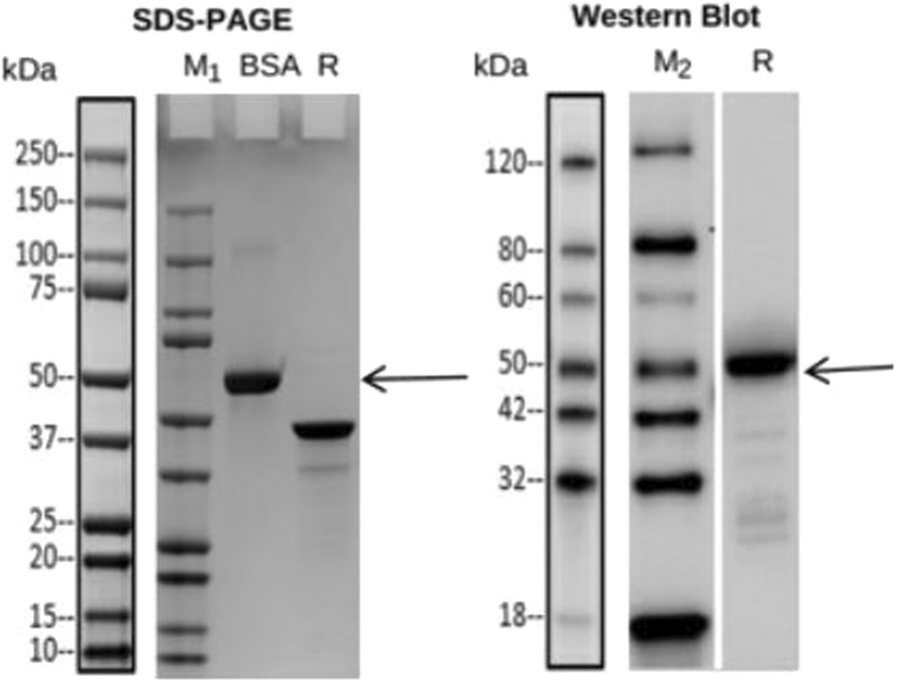
Expression of SARS-CoV-2 N antigen using wheat germ cell free system confirmed by SDS–PAGE and Western blot (anti-His tag, Abcam ab18184); M_1_: protein marker, Bio-Rad, Cat No. 1610374S; M_2_: protein marker, GenScript, Cat. No. M00673; BSA: 2.00 µg; R: reducing condition. The expected band size is ~ 46 kDa

The amplification of the N gene using specific primers by RT-PCR resulted in a 1200 bp fragment that was cloned into an expression vector. Sanger sequencing confirmed the correct insertion and orientation of the antigen sequence within the plasmid clones. The protein was confirmed using SDS–PAGE analysis and western blotting at the expected size of 46 kDa (Fig. 2).

### Hybridoma selection for mAbs production

**Fig. 3:**
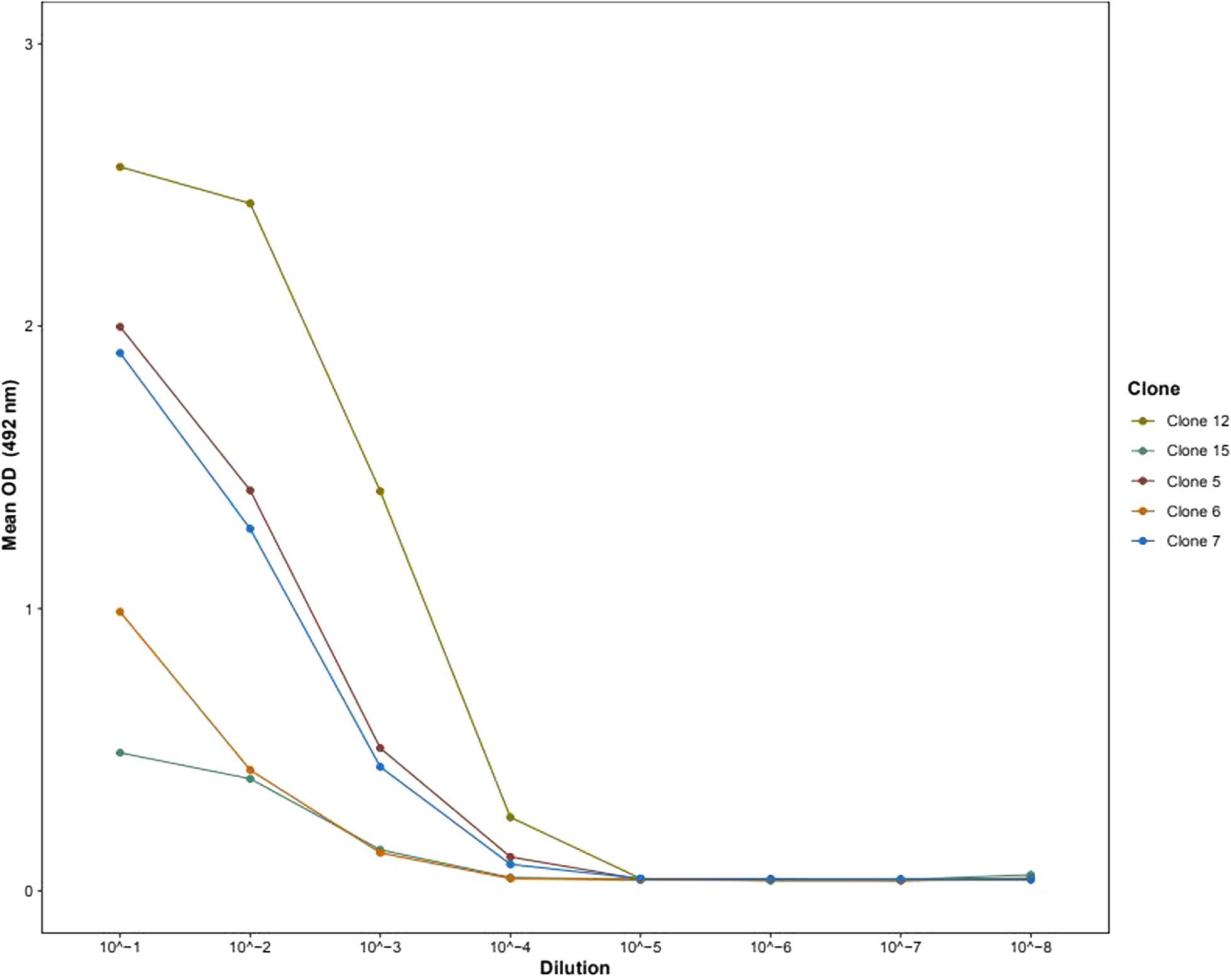
Evaluation of antigen binding affinities of selected hybridoma clones using indirect ELISA. Clone 12 (3H1–2) exhibited the highest binding affinity among the five clones

Among the 20 stable hybridomas generated, five clones (5, 6, 7, 12, and 15) were subcloned to select the most reactive hybridoma for mAb production. This selection was based on their binding affinities to the target antigen. The average OD values at 492 nm ranged from approximately 0.1 to 2.5 across serial dilutions (Fig. 3). Clone 12 exhibited the strongest binding affinity, maintaining higher OD values across the dilutions, with a peak OD of 2.5 at the initial dilution (10^1^). Clone 15 followed with slightly lower binding strength, while clones 5, 6, and 7 demonstrated progressively weaker affinities (Fig. 3). Based on these findings, clone 12 (3H1–2) was selected for monoclonal antibody production due to its superior performance in antigen binding.

### Reactivity screening of the study mAb and commercial mAb

**Fig. 4:**
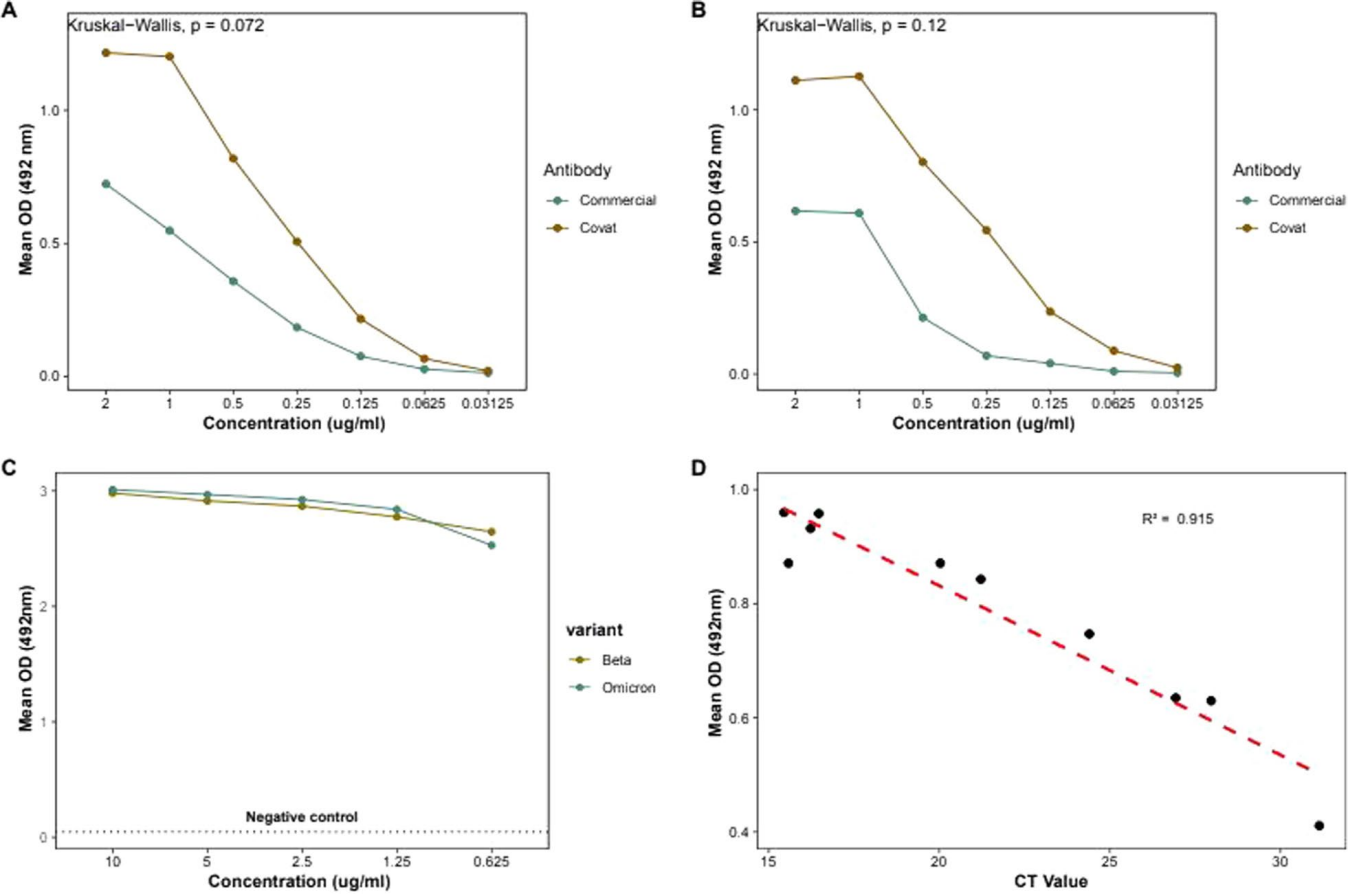
**A**, **B** Titration curves showing the binding efficiency of two antibodies; Covat (study mAb from clone 12–3H1–2) and Commercial mAb (2G4–H1–B2–C3, Cat No. 130–10836) against commercial antigen (Nagasaki, DIA-5028) and study recombinant antigen, respectively. Antibody concentrations range from 2.0 to 0.03125 µg/ml. Statistical comparison using the Kruskal–Wallis test showed no significant difference in binding (p = 0.072 and p = 0.12 for panels A and B, respectively)** C** Binding activity of varying concentrations of our monoclonal antibody against SARS-CoV-2 beta and omicron variants from Kenya and Japan, respectively. The monoclonal antibody with decreasing concentrations (10 µg/ml, 5 µg/ml, 2.5 µg/ml, 1.25 µg/ml and 0.625 µg/ml) showed strong and consistent signal against the virus infected culture fluid (ICF) both Beta (3.45 × 10^5^ PFU/mL) and Omicron (3.45 × 10^5^ PFU/mL) SARS-CoV-2 variants, with minimal reduction in optical density.** D** Correlation between RT-qPCR cycle threshold (CT) values and ELISA absorbance at 492 nm. An inverse relationship was observed (R^2^ = 0.915), indicating high assay sensitivity.

We evaluated the reactivity of this study and commercial mAb against both commercial and our antigen, CoVAT. Our mAb showed a higher reactivity against both antigens, but there was no statistical difference in the binding affinities of both mAbs (Fig. 4A, b). Using sandwich ELISA, we checked the reactivity of our mAb against Beta (B. 1.351) and Omicron (B.1.1.529) SARS-CoV-2 variants obtained from Kenya and Japan, respectively. The mAb effectively detected both variants, with the OD remaining high and stable across dilutions, suggesting strong cross-variant binding (Fig. 4C).

### Clinical performance of the study mAb

We further evaluated the ability of this study mAb to detect SARS-CoV-2 using clinical nasopharyngeal samples with confirmed SARS-CoV-2 infection across a range of RT-PCR Ct values. A clear inverse correlation was observed between the Ct values and ELISA absorbance at 492 nm (Fig. 4D), indicating higher antigen detection in samples with lower Ct values. The detected Ct values ranged between 15.5 and 31.14 and explained 91.5% of the data between the Ct values and absorbance measurements, indicating the study mAb performs optimally in samples with higher viral loads but also detect samples with lower viral loads. The findings underscore the utility of the study mAb in antigen detection-based platforms, especially in identifying SARS-CoV-2 infection samples with either high or low viral loads during any phase of an outbreak.

### mAb specificity screening against other respiratory viruses

**Fig. 5:**
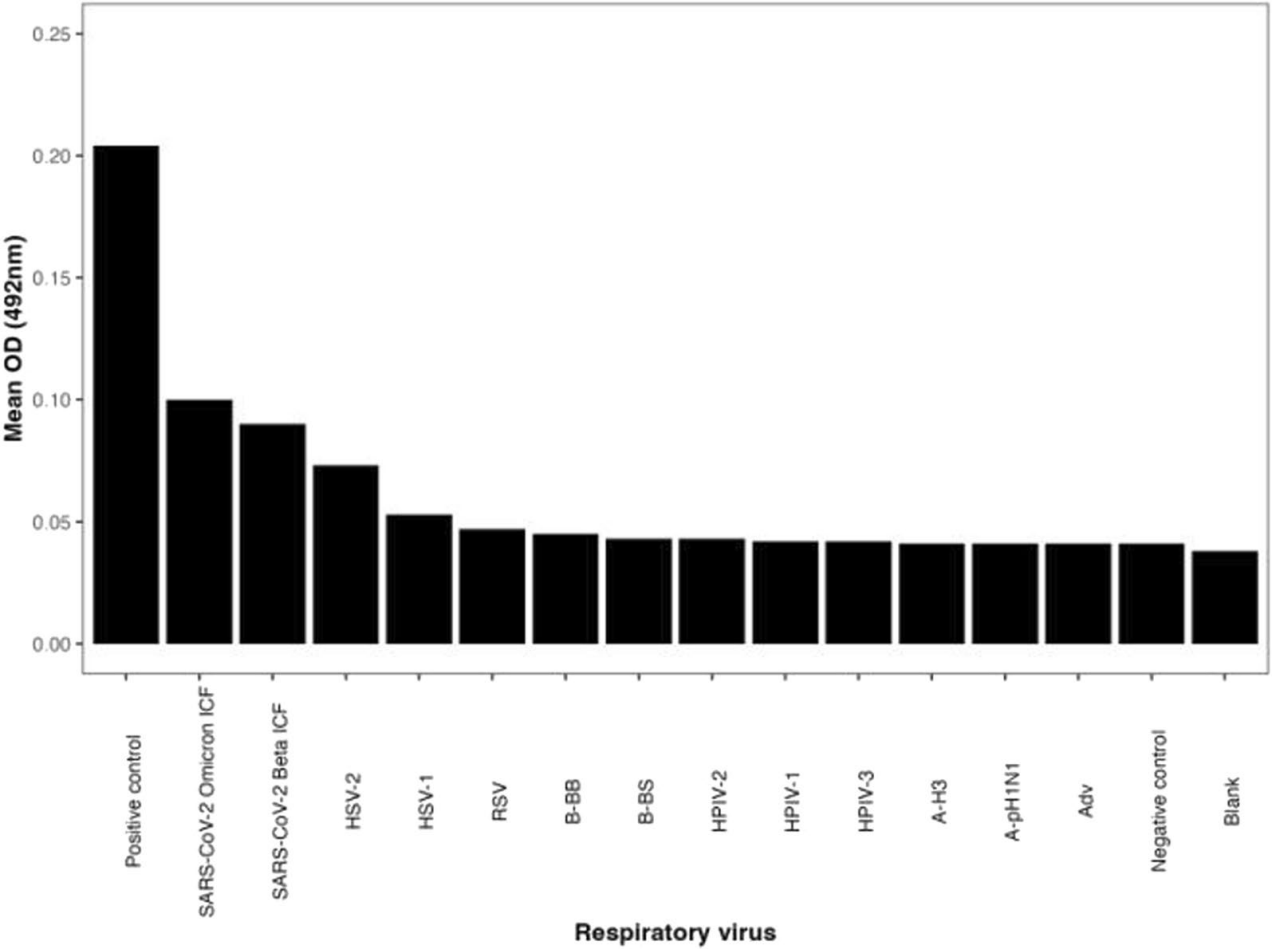
Specificity of monoclonal antibodies against other respiratory viruses: B-BB; influenza B virus/Yamagata lineage, B-BS; influenza B virus/Victoria lineage, A-H3; influenza A virus type H3 N2, RSV; respiratory syncytial virus, HSV-1/2; herpes simplex virus types 1 and 2, A-pH1 NI; influenza A virus type H1 N1, Adv; adenovirus; HPIV-1,2,3; human parainfluenza virus, PC; positive control (recombinant SARS-CoV-2 N protein)

The mean absorbance values for Human parainfluenza virus (HPIV-1,2,3), Adenovirus (Adv), A-pH1 N1—influenza A virus type H1 N1, A-H3—influenza A virus type H3 N2, Influenza B virus (BS), B/Yamagata lineage, Influenza B virus (BB), B/Victoria lineage, Respiratory syncytial virus (RSV), Herpes simplex virus (HSV type 1/2), SARS-CoV-2 Beta and Omicron mutants was obtained. The positive control was our recombinant N antigen which exhibited the highest absorbance. The non-target viruses showed low absorbance, as shown in Fig. 5. SARS-CoV-2 beta and omicron variants showed higher reactivity compared to the other respiratory viruses.

## Discussion

In this study, we leveraged WGCFS to generate SARS-CoV-2 N recombinant protein which could be used for the active detection of SARS-CoV-2. WGCFS is a powerful tool that surpasses the traditional prokaryotic translation system. It can be utilized for large-scale production of proteins that are soluble and retain their native conformation, mimicking the structure of the target antigen encountered by the immune system [28–30]. These benefits highlight the suitability of WGCFS to produce quality antigens that can be used to immunize animals for production of precise and reliable mAbs. This system has been used to express proteins for drug development [23], vaccine targets [26], as well as for monoclonal antibody production [10]. Our mAb demonstrated high reactivity against our SARS-CoV-2 N antigen as well as the commercial N protein (Fig. 4A, B. The higher reactivities observed in our SARS-CoV-2 antigen may be attributed to similarities in conformational epitopes recognized from the mAbs, while the reactivities in the commercial antigen are likely associated with the conserved nature of conformational epitopes present in the N protein.

SARS-CoV-2 evolution is primarily driven by point mutations and recombination resulting in new lineages and variants of concern defined by their reduced effectiveness to public health interventions [5]. The major VOCs are Beta (B.1.315), Alpha (B.1.1.7), Delta (B.1.617.2), Gamma (P.1), and Omicron (B.1.1.529) [31]. While the spike protein has been undergoing changes due to immune pressure, mutations have also been reported in the nucleocapsid protein [5]. This raises a fundamental question on whether the currently generated monoclonal antibodies will be able to detect active infections or result in false negative cases. These mutations have impacted both molecular and serological diagnosis of SARS-CoV-2. S gene mutations have necessitated regular validation of commercial RT-PCR kits to prevent false negatives, while mutations in N and RdRp genes have shown to reduce assay sensitivity. Mutations that alter epitope structure prevent mAbs from binding effectively. This can lead to reduced sensitivity making polyclonal antibodies a more reliable option [32]. However, monoclonal antibodies targeting conserved regions of the N gene could help minimize false negatives by maintaining their ability to bind despite viral mutations. By focusing on stable regions of the N gene, mAbs could provide more consistent results across various SARS-CoV-2 variants, enhancing diagnostic accuracy.

Our generated monoclonal antibody was able to detect both the Beta (B.1.315) and Omicron (B.1.1.529) variants with the omicron variant showing better reactivity values to the beta variant from ICF (Fig. 4C). The detection of both variants reinforces the dependability of using the N antigen for SAR-CoV-2 diagnostic test. Previous developed monoclonal antibody using the N protein were able to detect both the alpha variant with mutations D3L, R203 K, G204R, and S235 F and the delta variant with mutations D63G, R203M, D377Y, and R385 K as well as the wild type, beta and omicron variant [33]. These results indicate that the presence of these mutations may not prevent the epitopes from binding on the monoclonal antibody. Our mAb demonstrated the ability to detect SARS-CoV-2 antigen from nasopharyngeal swab clinical samples across a range of Ct values in ELISA-based detection (Fig. 4D). This capability is important considering the phases of any infection are associated with varying levels of viral loads, with the early and late phase of the SARS-CoV-2 wave being linked with high Ct values. By enabling timely detection throughout the course of infection, our mAb could contribute to public health interventions.

We evaluated the specificity of our mAb against other respiratory viruses. The binding affinity of our recombinant N antigen, which was used as the positive control exhibited the highest absorbance, confirming the validity of the assay. The other non-target viruses including Human parainfluenza virus (HPIV-1,2,3), Adenovirus (Adv), A-pH1 N1—influenza A virus type H1 N1, A-H3—influenza A virus type H3 N2, Influenza B virus (BS), B/Yamagata lineage, Influenza B virus (BB), B/Victoria lineage, Respiratory syncytial virus (RSV), and Herpes simplex virus (HSV type 1), did not indicate cross-reactivity with our monoclonal antibody. Although we noted some potential reactivity between our mAb and HSV-2, this may be attributed to epitope cross-reactivity. A study reported that mAbs produced against SARS-CoV-2 N antigen showed cross-reactivity with parts of SARS-CoV but not on other coronaviruses [34]. Notably, in this study, our monoclonal antibody successfully bound to SARS-CoV-2 beta and omicron variants. This indicates that the selected epitope remains partially conserved across different SARS-CoV-2 lineages, supporting its potential use in variant-inclusive diagnostics. A similar study reported low to negative reactivity of the study mAb to other coronaviruses including SARS-CoV-1 but strong reaction towards omicron lineages [35].

The limitation of this study is that we did not evaluate the mAb against other coronaviruses, such as SARS-CoV-1, MERS-CoV, and common cold coronaviruses, such as OC43, 229E, NL63, and HKU1. Assessing cross-reactivity with these viruses would provide a more comprehensive understanding of our mAbs specificity and potential limitations in distinguishing SARS-CoV-2 from related coronaviruses. In addition, while a limited number of clinical samples were tested to assess the diagnostic performance of the mAbs, broader evaluation using a larger and more diverse set of clinical specimens is still needed. This would help establish the sensitivity, specificity, and the clinical applicability of the antibodies across different populations and viral variants.

Despite these limitations, the high reactivity pair of our mAb and recombinant antigen suggests potential for their use in development of lateral flow antigen (LFA) kits for point of care use, which are currently under development. Furthermore, our mAbs cab be applied in therapeutic development and environmental or clinical surveillance platforms.

## Conclusion

This study demonstrated the successful use of the WGCFS to express SARS-CoV-2 N antigen in resource-limited setting. We generated mAb against SARS-CoV-2 in mice which showed high reactivity against our recombinant protein, commercial (Nagasaki) antigen, SARS-CoV-2 beta and omicron variants. Our mAb showed comparable reactivity to that of a commercially available antibody, reinforcing its diagnostic potential. While no cross-reactivity was observed with common respiratory viruses, further validation is needed to assess potential cross-reactivity with other coronaviruses and to confirm performance in large-scale clinical testing. These findings support its potential for use in the development of LFA kit which is ongoing as well as therapeutic development and clinical or environmental surveillance platforms.

## Data Availability

No datasets were generated or analysed during the current study.

## Abbreviations

ACE-2: Angiotensin-converting enzyme 2
Adv: Adenovirus
A-H3: Influenza A virus
A-pH1 N1: Influenza A virus type H1N1
ATP: Adenosine triphosphate
BB: Influenza B virus/Victoria lineage
BS: Influenza B virus/Yamagata lineage
BSA: Bovine serum albumin
COVID-19: Coronavirus 2019
CT: Cycle threshold
DPP4: Dipeptidyl peptidase 4
ELISA: Enzyme linked immuno-assays
FDA: Food and drug Administration
GTP: Guanosine triphosphate
HAT: Hypoxanthine aminopterin and thymine
HPIV: Human parainfluenza virus
HSV: Herpes simplex virus
ICF: Infected fluid culture
LFA: Lateral flow antigen
mAb: Monoclonal antibody
MAFFT: Multiple alignment using Fast Fourier Transform
MERS-CoV: Middle East Respiratory Syndrome Coronavirus
NCBI: National centre for biotechnology information
NMAbs: Neutralizing monoclonal antibodies
OPD: O-phenyl diamine
PBS-T: Phosphate buffered saline with tween 20
PEG: Polyethylene glycol
RBD: RNA binding domain
RSV: Respiratory syncytial virus
RT-qPCR: Reverse transcription real-time polymerase chain reaction
SARS-CoV-1: Severe acute respiratory syndrome coronavirus
SARS-CoV-2: Severe acute respiratory syndrome coronavirus-2
SDS–PAGE: Sodium dodecyl-sulfate–polyacrylamide gel electrophoresis
WGCFS: Wheat germ cell-free system

## References

[1] Weiss SR, Navas-Martin S. Coronavirus pathogenesis and the emerging pathogen severe acute respiratory syndrome coronavirus. Microbiol Mol Biol Rev. 2005;69:635–64. 16339739 10.1128/MMBR.69.4.635-664.2005 PMC1306801

[2] Majumder J, Minko T. Recent developments on therapeutic and diagnostic approaches for COVID-19. AAPS J. 2021. 10.1208/s12248-020-00532-2. 33400058 10.1208/s12248-020-00532-2 PMC7784226

[3] Forchette L, Sebastian W, Liu T. A comprehensive review of COVID-19 virology, vaccines, variants, and therapeutics. Curr Med Sci. 2021;41:1037–51. 34241776 10.1007/s11596-021-2395-1 PMC8267225

[4] Raman R, Patel KJ, Ranjan K. COVID-19: Unmasking Emerging SARS-CoV-2 Variants, Vaccines and Therapeutic Strategies. Biomolecules. 2021;11. 10.3390/biom11070993 PMC8301790 34356617

[5] Alsuwairi FA, Alsaleh AN, Alsanea MS, Al-Qahtani AA, Obeid D, Almaghrabi RS, et al. Association of SARS-CoV-2 Nucleocapsid Protein Mutations with Patient Demographic and Clinical Characteristics during the Delta and Omicron Waves. Microorganisms. 2023;11. 10.3390/microorganisms11051288 PMC10224071 37317262

[6] Harvey WT, Carabelli AM, Jackson B, Gupta RK, Thomson EC, Harrison EM, et al. SARS-CoV-2 variants, spike mutations and immune escape. Nat Rev Microbiol. 2021;19:409–24. 34075212 10.1038/s41579-021-00573-0 PMC8167834

[7] Mutantu PN, Ngwe Tun MM, Nabeshima T, Yu F, Mukadi PKT, et al. Tanaka Development and evaluation of quantitative immunoglobulin g enzyme-linked immunosorbent assay for the diagnosis of coronavirus disease,using truncated recombinant nucleocapsid protein as assay antigen. Int J Environ Res Public Health. 2019;2021:18. 10.3390/ijerph18189630 PMC8469721 34574555

[8] Safiabadi Tali SH, LeBlanc JJ, Sadiq Z, Oyewunmi OD, Camargo C, Nikpour B, et al. Tools and techniques for severe acute respiratory syndrome coronavirus 2 (SARS-CoV-2)/COVID-19 detection. Clin Microbiol Rev. 2021;34:1–63. 10.1128/CMR.00228-20 PMC8142517 33980687

[9] Yamayoshi S, Sakai-Tagawa Y, Koga M, Akasaka O, Nakachi I, Koh H, et al. Comparison of Rapid Antigen Tests for COVID-19. Viruses. 2020. 10.3390/v12121420. 33322035 10.3390/v12121420 PMC7764512

[10] Yamaoka Y, Miyakawa K, Jeremiah SS, Funabashi R, Okudela K, Kikuchi S, et al. Highly specific monoclonal antibodies and epitope identification against SARS-CoV-2 nucleocapsid protein for antigen detection tests. Cell reports Med. 2021;2: 100311. 10.1016/j.xcrm.2021.100311 PMC8126173 34027498

[11] Focosi D, McConnell S, Casadevall A, Cappello E, Valdiserra G, Tuccori M. Monoclonal antibody therapies against SARS-CoV-2. Lancet Infect Dis. 2022;22:e311–26. 35803289 10.1016/S1473-3099(22)00311-5 PMC9255948

[12] Zhang Y, He Y, Li L, Liang S, Yan M, Ren D, et al. Development and characterization of an HPV18 detection kit using two novel HPV18 type-specific monoclonal antibodies. Diagn Pathol. 2018;13:55. 30115088 10.1186/s13000-018-0727-7 PMC6097307

[13] Ramathudi-Dunbar L, Awosanya E, Charles SB, Chitsungo E, Moustapha Boukary CR, Nwankpa N, et al. Development and Evaluation of Monoclonal Antibodies against CBPP Antigen with the End Goal of Developing an ELISA Kit. Vet Med Int. 2024;2024:6901355. 38746871 10.1155/2024/6901355 PMC11093687

[14] Qriouet Z, Cherrah Y, Sefrioui H, Qmichou Z. Monoclonal Antibodies Application in Lateral Flow Immunochromatographic Assays for Drugs of Abuse Detection. Molecules. 2021. 10.3390/molecules26041058. 33670468 10.3390/molecules26041058 PMC7922373

[15] Castelli MS, McGonigle P, Hornby PJ. The pharmacology and therapeutic applications of monoclonal antibodies. Pharmacol Res Perspect. 2019;7: e00535. 31859459 10.1002/prp2.535 PMC6923804

[16] Majidi J, Barar J, Baradaran B, Abdolalizadeh J, Omidi Y. Target therapy of cancer: Implementation of monoclonal antibodies and nanobodies. Hum Antibodies. 2009;18. 10.3233/HAB-2009-0204 19729803

[17] El Abd Y, Tabll A, Smolic R, Smolic M. Mini-review: The market growth of diagnostic and therapeutic monoclonal antibodies - SARS CoV-2 as an example. Hum Antibodies. 2022;30:15–24. 34958012 10.3233/HAB-211513

[18] Xiang R, Wang Y, Wang L, Deng X, Huo S, Jiang S, et al. Neutralizing monoclonal antibodies against highly pathogenic coronaviruses. Curr Opin Virol. 2022;53: 101199. 35038651 10.1016/j.coviro.2021.12.015 PMC8716168

[19] Jiang L, Wang N, Zuo T, Shi X, Poon K-MV, Wu Y, et al. Potent neutralization of MERS-CoV by human neutralizing monoclonal antibodies to the viral spike glycoprotein. Sci Transl Med. 2014;6:234ra59. 24778414 10.1126/scitranslmed.3008140

[20] Ying T, Prabakaran P, Du L, Shi W, Feng Y, Wang Y, et al. Junctional and allele-specific residues are critical for MERS-CoV neutralization by an exceptionally potent germline-like antibody. Nat Commun. 2015;6:8223. 26370782 10.1038/ncomms9223 PMC4571279

[21] Peng Y, Liu Y, Hu Y, Chang F, Wu Q, Yang J, et al. Monoclonal antibodies constructed from COVID-19 convalescent memory B cells exhibit potent binding activity to MERS-CoV spike S2 subunit and other human coronaviruses. Front Immunol. 2022;13:1056272. 36618428 10.3389/fimmu.2022.1056272 PMC9813381

[22] Hwang Y-C, Lu R-M, Su S-C, Chiang P-Y, Ko S-H, Ke F-Y, et al. Monoclonal antibodies for COVID-19 therapy and SARS-CoV-2 detection. J Biomed Sci. 2022;29:1. 34983527 10.1186/s12929-021-00784-w PMC8724751

[23] Yamaoka Y, Jeremiah SS, Funabashi R, Miyakawa K, Morita T, Mihana Y, et al. Characterization and Utilization of Disulfide-Bonded SARS-CoV-2 Receptor Binding Domain of Spike Protein Synthesized by Wheat Germ Cell-Free Production System. Viruses. 2022. 10.3390/v14071461. 35891441 10.3390/v14071461 PMC9321213

[24] Kotani H, Matsuda KM, Yamaguchi K, Ono C, Kogo E, Ogawa K, et al. Diversity and Epitope Spreading of Anti-RNA Polymerase III Antibodies in Systemic Sclerosis: A Potential Biomarker for Skin and Lung Involvement. Arthritis Rheumatol (Hoboken, NJ). 2025;77:67–79. 10.1002/art.42975 PMC11684998 39219033

[25] Kanoi BN, Takashima E, Morita M, White MT, Palacpac NMQ, Ntege EH, et al. Antibody profiles to wheat germ cell-free system synthesized Plasmodium falciparum proteins correlate with protection from symptomatic malaria in Uganda. Vaccine. 2017;35:873–81. 28089547 10.1016/j.vaccine.2017.01.001

[26] Nagaoka H, Sasaoka C, Yuguchi T, Kanoi BN, Ito D, Morita M, et al. PfMSA180 is a novel Plasmodium falciparum vaccine antigen that interacts with human erythrocyte integrin associated protein (CD47). Sci Rep. 2019;9:5923. 30976034 10.1038/s41598-019-42366-9 PMC6459815

[27] Kanoi BN, Nagaoka H, Morita M, Tsuboi T, Takashima E. Leveraging the wheat germ cell-free protein synthesis system to accelerate malaria vaccine development. Parasitol Int. 2021;80: 102224. 33137499 10.1016/j.parint.2020.102224

[28] Takai K, Sawasaki T, Endo Y. Practical cell-free protein synthesis system using purified wheat embryos. Nat Protoc. 2010;5:227–38. 20134421 10.1038/nprot.2009.207

[29] The TK, System W-G-F. Curr Pharm Biotechnol. 2010;999:1–13.

[30] Harbers M. Wheat germ systems for cell-free protein expression. FEBS Lett. 2014;588:2762–73. 24931374 10.1016/j.febslet.2014.05.061

[31] Hossain A, Akter S, Rashid AA, Khair S, Alam ASMRU. Unique mutations in SARS-CoV-2 Omicron subvariants’ non-spike proteins: Potential impacts on viral pathogenesis and host immune evasion. Microb Pathog. 2022;170:105699. 35944840 10.1016/j.micpath.2022.105699 PMC9356572

[32] Thakur S, Sasi S, Pillai SG, Nag A, Shukla D, Singhal R, et al. SARS-CoV-2 mutations and their impact on diagnostics therapeutics and vaccines. Front Med. 2022;9: 815389. 10.3389/fmed.2022.815389 PMC8902153 35273977

[33] Lu R-M, Ko S-H, Chen W-Y, Chang Y-L, Lin H-T, Wu H-C. Monoclonal Antibodies against Nucleocapsid Protein of SARS-CoV-2 Variants for Detection of COVID-19. Int J Mol Sci. 2021. 10.3390/ijms222212412. 34830291 10.3390/ijms222212412 PMC8623253

[34] Wen Y, Guo W, Min Y, Zhong K, Zhang X, Xing X, et al. Patient-derived monoclonal antibodies to SARS-CoV-2 nucleocapsid protein N-terminal and C-terminal domains cross-react with their counterparts of SARS-CoV, but not other human betacoronaviruses. Front Immunol. 2023. 10.3389/fimmu.2023.1093709. 36798118 10.3389/fimmu.2023.1093709 PMC9927002

[35] Qiu H, Yuan X-Y, Holloway K, Wood H, Cabral T, Grant C, et al. Development and characterization of monoclonal antibodies recognizing nucleocapsid protein of multiple SARS-CoV-2 variants. Heliyon. 2024;10: e35325. 39170261 10.1016/j.heliyon.2024.e35325 PMC11336563

